# Solution Structure and Characterisation of the Human Pyruvate Dehydrogenase Complex Core Assembly

**DOI:** 10.1016/j.jmb.2010.03.043

**Published:** 2010-05-28

**Authors:** S. Vijayakrishnan, S.M. Kelly, R.J.C. Gilbert, P. Callow, D. Bhella, T. Forsyth, J.G. Lindsay, O. Byron

**Affiliations:** 1 Division of Molecular and Cell Biology, Faculty of Biomedical and Life Sciences, Davidson Building, University of Glasgow, Glasgow G12 8QQ, UK; 2 Division of Infection and Immunity, Faculty of Biomedical and Life Sciences, Glasgow Biomedical Research Centre, University of Glasgow, Glasgow G12 8TA, UK; 3 Division of Molecular and Cell Biology, Faculty of Biomedical and Life Sciences, Joseph Black Building, University of Glasgow, Glasgow G12 8QQ, UK; 4 Division of Structural Biology, Wellcome Trust Centre for Human Genetics, University of Oxford, Roosevelt Drive, Oxford OX3 7BN, UK; 5 EPSAM and ISTM Research Institutes, Keele University, Staffordshire ST5 5BG, UK; 6 Partnership for Structural Biology, Institut Laue Langevin, 6 rue Jules Horowitz, 38042 Grenoble, France; 7 Institute of Virology, University of Glasgow, Church Street, Glasgow G11 5JR, UK

**Keywords:** PDC, pyruvate dehydrogenase complex, OGDC, 2-oxoglutarate dehydrogenase complex, LD, lipoyl domain, SBD, subunit binding domain, CTD, C-terminal domain, PDB, Protein Data Bank, EM, electron microscopy, AUC, analytical ultracentrifugation, SAXS, small-angle X-ray scattering, SANS, small-angle neutron scattering, SV, sedimentation velocity, SE, sedimentation equilibrium, GFC, gel-filtration chromatography, HBM, hydrodynamic bead model, SAS, small-angle scattering, CTF, contrast transfer function, EDTA, ethylenediaminetetraacetic acid, EMBL, European Molecular Biology Laboratory, ILL, Institut Laue Langevin, pyruvate dehydrogenase complex, SAS, AUC, cryo-EM, GdmCl unfolding

## Abstract

Mammalian pyruvate dehydrogenase complex (PDC) is a key multi-enzyme assembly that is responsible for glucose homeostasis maintenance and conversion of pyruvate into acetyl-CoA. It comprises a central pentagonal dodecahedral core consisting of two subunit types (E2 and E3BP) to which peripheral enzymes (E1 and E3) bind tightly but non-covalently. Currently, there are two conflicting models of PDC (E2 + E3BP) core organisation: the ‘addition’ model (60 + 12) and the ‘substitution’ model (48 + 12). Here we present the first ever low-resolution structures of human recombinant full-length PDC core (rE2/E3BP), truncated PDC core (tE2/E3BP) and native bovine heart PDC core (bE2/E3BP) obtained by small-angle X-ray scattering and small-angle neutron scattering. These structures, corroborated by negative-stain and cryo electron microscopy data, clearly reveal open pentagonal core faces, favouring the ‘substitution’ model of core organisation. The native and recombinant core structures are all similar to the truncated bacterial E2 core crystal structure obtained previously. Cryo-electron microscopy reconstructions of rE2/E3BP and rE2/E3BP:E3 directly confirm that the core has open pentagonal faces, agree with scattering-derived models and show density extending outwards from their surfaces, which is much more structurally ordered in the presence of E3. Additionally, analytical ultracentrifugation characterisation of rE2/E3BP, rE2 (full-length recombinant E2-only) and tE2/E3BP supports the substitution model. Superimposition of the small-angle neutron scattering tE2/E3BP and truncated bacterial E2 crystal structures demonstrates conservation of the overall pentagonal dodecahedral morphology, despite evolutionary diversity. In addition, unfolding studies using circular dichroism and tryptophan fluorescence spectroscopy show that the rE2/E3BP is less stable than its rE2 counterpart, indicative of a role for E3BP in core destabilisation. The architectural complexity and lower stability of the E2/E3BP core may be of benefit to mammals, where sophisticated fine-tuning is required for cores with optimal catalytic and regulatory efficiencies.

## Introduction

Mitochondrial 2-oxoacid dehydrogenase complexes are a family of stable macromolecular machines (*M*_r _= 4–10 million) that serve as models for the study of protein–protein interactions, enzyme cooperativity and active-site coupling. Principal members include the pyruvate dehydrogenase complex (PDC), 2-oxoglutarate dehydrogenase complex (OGDC) and branched-chain 2-oxoacid dehydrogenase complex. The lipoamide prosthetic group [on E2 (dihydrolipoamide acetyltransferase) and E3BP (E3 binding protein) in PDC] assists all three complexes in their catalytic function via its ‘swinging-arm’ mechanism, visiting all the active sites during the multi-step reaction cycle.^1,2^ PDC links glycolysis with the tricarboxylic acid cycle, catalysing the irreversible decarboxylation of pyruvate to acetyl-CoA, the key committed step in carbohydrate utilisation in mammals.^3^ In recent years, PDC and OGDC defects have been implicated in several genetic and physiological disorders, including metabolic acidosis,^4–6^ diabetes^7–9^ and primary biliary cirrhosis;^10–12^ neurodegenerative conditions such as Alzheimer's disease;^13–16^ and other disorders linked to oxidative stress.^17^

PDC comprises multiple copies of three distinct enzymes: E1 (pyruvate decarboxylase), E2 and E3 (dihydrolipoamide dehydrogenase). In addition, eukaryotic PDC contains a unique subunit, termed E3BP,^18,19^ that has no known enzymatic function. The central core of PDC is made up of E2 (and E3BP in eukaryotes), forming either a 24-meric octahedron (in Gram-negative bacteria) or a 60-meric pentagonal dodecahedron (in eukaryotes and Gram-positive bacteria).^20,21^ Thus, E2 provides the structural and organisational framework upon which the assembly and function of the entire complex are dependent, in addition to its role in catalysis. The E2 core interacts with both E1 and E3 in a mutually exclusive fashion in bacteria,^2,22^ whereas in eukaryotes, E3BP specifically mediates stable E3 integration.^18,19^ Interestingly, the PDC of patients totally lacking E3BP displays 10–20% of wild-type activity and appears to retain a residual affinity for E3.^23^

**Fig. 1:**
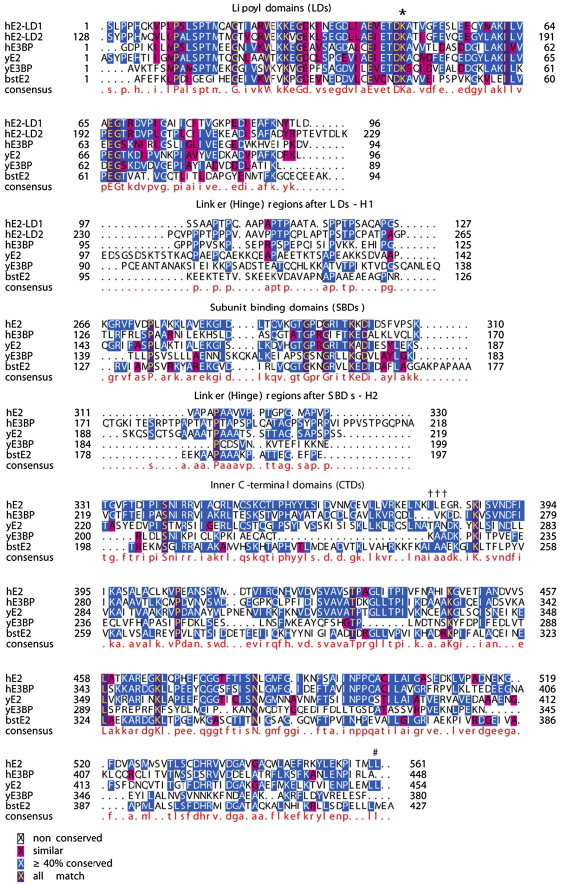
Sequence alignment of eukaryotic E2 and E3BP. ClustalW (http://www.ebi.gla.ac.uk/clustalw) alignment of the amino acid sequences of E2 and E3BP [human (h), yeast (y) and *B. stearothermophilus* (bst)]. LDs, linker regions (H1 and H2), SBDs and inner CTDs of human E2 and E3BP, along with the consensus sequence (red), are indicated. Sequence numbers on the right indicate the end of domains, and the lipoylation site (⁎) and key residues mediating the ‘ball-and-socket’ interaction (# and †) between trimeric units are also indicated. The image was created using TEXshade (http://www.ctan.org/tex-archive/help/Catalogue/entries/texshade.html).

Human E2 and E3BP have similar modular domain organisations. They both comprise highly flexible N-terminal lipoyl domains (LDs; two on E2 and one on E3BP), followed by a subunit binding domain (SBD) and a C-terminal domain (CTD) that is involved in core assembly (Fig. 1). These modular domains are connected by Ala-Pro-rich linkers that confer mobility to the peripherally extended LDs such that their covalently bound lipoamide cofactors can serve as substrates for all three constituent enzymes in turn during catalysis. PDC is acutely regulated by tightly bound kinases (phosphoinositide-dependent protein kinases) and loosely bound phosphatases (pyruvate dehydrogenase phosphatases) that act in concert to control its activity state.^24^ Phosphoinositide-dependent protein kinase is a major drug target, as activation of PDC by limiting phosphorylation can alleviate impaired carbohydrate metabolism in severe diabetes.

Several structures of the individual domains (LD, SBD and CTD) of E2 PDC have been solved to high resolution.^25–29^ Of most relevance to this study are the crystal structures of the truncated E2 CTD [tE2 (truncated E2-only PDC core)] of *Azotobacter vinelandii*^30^ and *Bacillus stearothermophilus*.^28^ The high-resolution crystal structure of the truncated core from *B. stearothermophilus* [Protein Data Bank (PDB) ID 1B5S], in particular, provides clear insights into its subunit organisation, with the basic building blocks (namely, truncated E2 trimers) located at the 20 vertices of the icosahedron^28^ interconnected by 30 flexible bridges that enable the core to ‘breathe’, as evidenced by a size variability of 20%.^31^ A recent cryo electron microscopy (EM) reconstruction of truncated recombinant human E2 has also been determined.^32^ However, there is as yet no structure for the human E2/E3BP assembly, probably owing to its massive size, significant N-terminal flexibility and subunit heterogeneity. Moreover, the precise subunit composition and arrangement of E2 and E3BP subunits around its pentagonal dodecahedral core are still unclear, as is the stoichiometry of E1 and E3 binding.

Previous densitometry and radiolabelling studies of purified bovine E2/E3BP indicated the presence of 12 E3BP molecules per core, suggestive of an icosahedral E2/E3BP assembly comprising 60 copies of E2 and 12 copies of E3BP.^33^ Similarly, Maeng *et al.* showed the binding of 12–15 E3BP molecules to the E2 core of *Saccharomyces cerevisiae* PDC.^34^ Comparative cryo-EM studies of truncated yeast E2 and E2/E3BP cores indicated the localisation of 12 E3BP molecules to the pentagonal faces, giving rise to the 60E2 + 12E3BP ‘addition model’.^35,36^ However, the recent work of Hiromasa *et al.* based on analytical ultracentrifugation (AUC) and small-angle X-ray scattering (SAXS) has led to the development of an alternative ‘substitution model’ wherein 12 E2 polypeptides are replaced by 12 E3BP subunits, resulting in a 48E2 + 12E3BP core assembly.^37^ Variation in the subunit organisation of the yeast and human PDC cores has been attributed to the dissimilarity of their E3BP sequences (Fig. 1).^35,37^ Recently, a second version of the ‘substitution model’ has proposed a 40E2 + 20E3BP composition based on isothermal titration calorimetry and AUC data.^38^

In the absence of detailed structural information, our current knowledge of the architecture, subunit organisation and functional properties of the human E2/E3BP core assembly remains limited. Moreover, no solution structures for the full-length recombinant or native mammalian core assemblies are available at present. In this context, the specific aims of this study were as follows:

(1) To employ SAXS and small-angle neutron scattering (SANS) technologies to obtain the first low-resolution structures of full-length recombinant human and native bovine E2/E3BP cores and the truncated human E2/E3BP equivalent. This will permit (a) direct comparison between them, and (b) assessment of their similarity to the published crystal structure of *B. stearothermophilus* tE2.
(2) To test the hypothesis that the human E2/E3BP core assembles via a ‘substitution’—and not ‘addition’—mechanism.
(3) To undertake a parallel cryo-EM evaluation of the recombinant full-length E2/E3BP core in the presence or in the absence of bound E3 (a) to make a direct comparison with the equivalent SAXS/SANS structures and, in so doing, test the hypothesis that cryo-EM imaging misses key features that can be detected by SAXS studies, and (b) to analyse the possible effects of E3 binding on the organisation of the peripherally located E3BP-linked SBDs and LDs.
(4) To determine for rE3BP its molecular mass and propensity for self-association (by AUC) and to assess its influence on core stability by monitoring differences between the profiles of GdmCl-induced E2 core and E2/E3BP core dissociation using circular dichroism (CD) and tryptophan fluorescence spectroscopy. In so doing, the hypothesis that E3BP destabilises the E2/E3BP core assembly will be tested.

## Results

### AUC analysis of recombinant cores rE2/E3BP, rE2 and tE2/E3BP

**Fig. 2:**
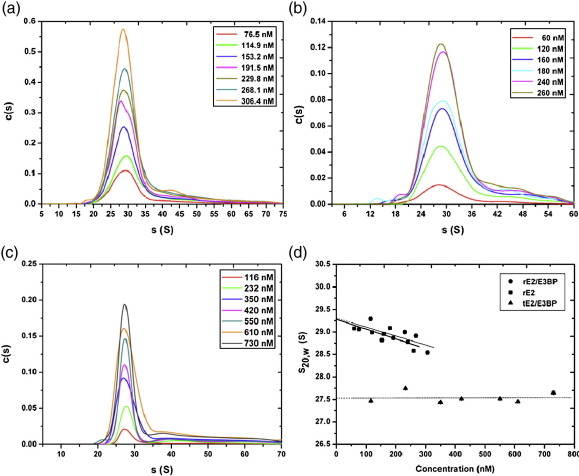
SV analysis of rE2/E3BP, rE2 and tE2/E3BP. *c*(*s*) distributions derived via SEDFIT from SV interference data collected over a range of sample concentrations for (a) rE2/E3BP (*s*_20,w_^0^ = 29.3 ± 0.04 S), (b) rE2 (*s*_20,w_^0^ = 29.3 ± 0.02 S) and (c) tE2/E3BP (*s*_20,w_^0^ = 27.5 ± 0.03 S). (d) Determination of the concentration-independent sedimentation coefficient for the main species. Error bars are shown but are not clearly visible owing to their small size.

The hydrodynamic and thermodynamic behaviour of the recombinant PDC cores was determined via sedimentation velocity (SV) and sedimentation equilibrium (SE) AUC. SV of rE2/E3BP (recombinant full-length PDC core; Fig. 2a), rE2 (recombinant full-length E2-only PDC core; Fig. 2b) and tE2/E3BP (recombinant truncated PDC core; Fig. 2c) revealed a main species whose sedimentation coefficient is consistent with that of a 60-meric core. Additionally, a trailing edge corresponding to a small amount (≤ 5% of total) of high-molecular-mass species was also observed. Weight-average sedimentation coefficients *s*_w_ were determined to be 29 and 43 S (rE2/E3BP), 28.5 and 47 S (rE2), and 27 and 38 S (tE2/E3BP) by integration of each peak in *c*(*s*) analysis. While the *s*_w_ of the major peak for rE2/E3BP (29 S) compares favourably with the sedimentation coefficient of 31.8 S obtained by Hiromasa *et al.*,^37^ the *s*_w_ of rE2 (28.5 S) is slightly lower than the value (36.0 S) they reported^37^ and may reflect the different approaches employed in sample preparation and data analysis. Finite-element analysis with the non-interacting discrete species model in SEDFIT yielded sedimentation coefficients for all three cores (rE2/E3BP, rE2 and tE2/E3BP) at all experimental concentrations. These were corrected to standard conditions and extrapolated to infinite dilution, giving concentration-independent sedimentation coefficients *s*_20,w_^0^ (Fig. 2d) of 29.3 ± 0.04 S (rE2/E3BP), 29.3 ± 0.02 S (rE2) and 27.5 ± 0.03 S (tE2/E3BP). Interestingly, the *c*(*s*) profile and sedimentation coefficient of tE2/E3BP (27.5 S; Fig. 2c) are comparable with SV data for full-length rE2/E3BP (29.3 S; Fig. 2a) and rE2 (29.3 S; Fig. 2b). Therefore, it can be inferred that the decrease in particle radius (and, as a consequence, the decrease in frictional drag) of tE2/E3BP is offset by the decrease in its molecular mass; thus, its sedimentation behaviour remains largely unchanged, compared with the full-length cores. The observed tail at higher *s* may correspond to the presence of aggregates or a small fraction of putative dimer that was persistent in all SV runs and has been observed previously.^37,39^ However, it was not possible to improve fits to the data with a monomer–dimer model; thus, the dimer, if real, may be irreversible. Moreover, the peaks corresponding to the intact 60-meric cores are quite broad and probably reflect inherent heterogeneity due to variation in the size of E2/E3BP cores, possibly as a result of the ‘breathing’ phenomenon observed previously^31^ (see Discussion).

Frictional ratios (*f*/*f*_0_) were derived from *s*_0_/*s*_20,w_^0^ (i.e., the ratio of the sedimentation coefficient of an anhydrous sphere of mass and volume equal to the core in question to the experimentally measured sedimentation coefficient). The resultant values (2.69, 2.79 and 1.73 for rE2/E3BP, rE2 and tE2/E3BP, respectively; Table 1) were consistent with the values obtained from *c*(*s*) analysis of SV data. *f*/*f*_0_ reflects experimental hydrodynamic hydration and deviation from sphericity. The large values of *f*/*f*_0_ for the full-length cores indicate structures with either significant deviation from spherical symmetry and/or significant hydration. The frictional ratios of rE2/E3BP and rE2 are almost indistinguishable from each other, but both are significantly greater than that of tE2/E3BP. This reflects the increase in deviation from the sphericity of the full-length cores due to their extended N-terminal domains.

The diameter of tE2/E3BP (*D*_s_ = 27.4 nm, derived from its hydrodynamic radius) is larger than the previously reported values of the average diameter, ranging from 21 to 24 nm.^40–42^ However, this elevated value is well within the 20% variability attributed to the ‘breathing’ of the inner core.^31^ At its N terminus, tE2/E3BP includes the 19-amino-acid and 30-amino-acid segments of the inner linker regions preceding the CTDs of E2 and E3BP, respectively. These extra linker regions will be located outside of the dodecahedron and will contribute to the hydrodynamic diameter of tE2/E3BP.

**Fig. 3:**
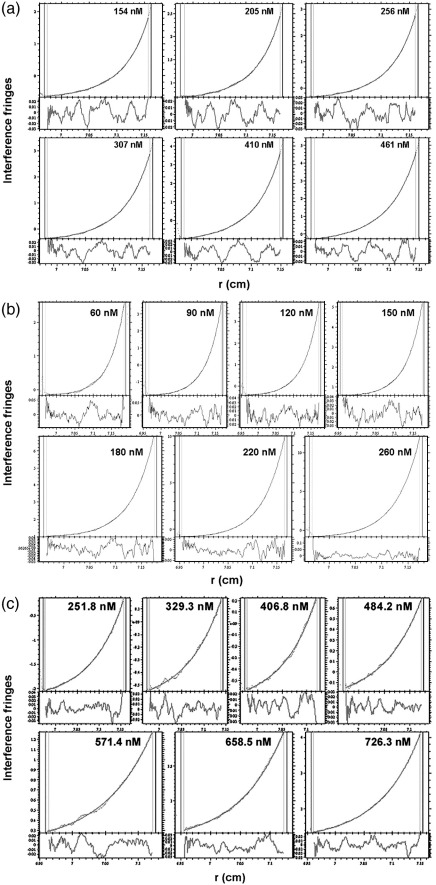

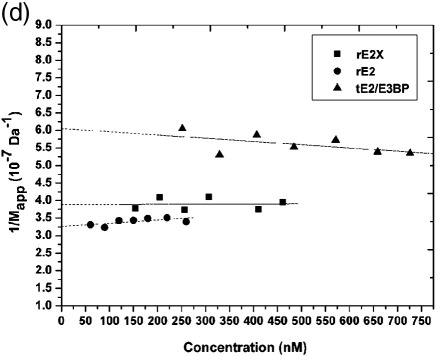
SE analysis of rE2/E3BP, rE2 and tE2/E3BP. Best fits (smooth lines) to the SE interference data (dotted lines) for (a) rE2/E3BP, (b) rE2 and (c) tE2/E3BP at 3000 rpm using a two-species model in SEDPHAT. Residual plots are shown beneath each fit. *M*_app_ was determined at each sample concentration. (d) Extrapolation of the 1/*M*_app_-*versus*-concentration plot of the predominant species to zero yielded the molecular mass *M*_w_^0^ independent of concentration for rE2/E3BP (2.56 MDa), rE2 (3.06 MDa) and tE2/E3BP (1.65 MDa).

**Fig. 4:**
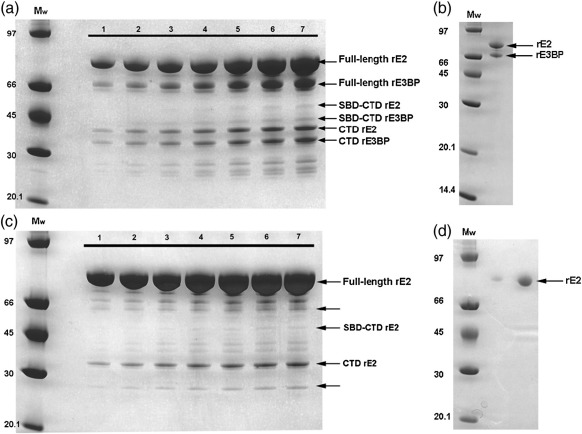
SDS-PAGE of post-SE rE2/E3BP and rE2 samples. Post-SE samples of (a) rE2/E3BP (lanes 1–7) and (c) rE2 (lanes 1–7) were analysed by SDS-PAGE and show several bands relating to proteolytic products (arrows) of rE2 and rE3BP. The different lanes represent the various concentrations of samples used. Freshly purified (b) rE2/E3BP and (d) rE2 are shown for comparison. Molecular masses of marker proteins (*M*_w_) are indicated in kilodaltons.

Global analysis of SE data by SEDPHAT^43,44^ with a single-species model yielded very poor fits that were greatly improved upon using a two-species model, giving molecular masses of 2.48 and 5.71 MDa (rE2/E3BP; Fig. 3a) and 2.78 and 4.59 MDa (rE2; Fig. 3b) for the predominant (∼ 90% of total) and minor (∼ 10% of total) species, respectively. The molecular mass of the predominant species independent of concentration *M*_w_^0^ (Fig. 3d) was determined to be 2.57 ± 0.24 MDa (rE2/E3BP) and 3.06 ± 0.25 MDa (rE2), lower than their predicted molecular masses of 3.55 MDa (based on the 48E2 + 12E3BP model) and 3.74 MDa, respectively. This discrepancy could reflect the formation of incomplete cores or proteolytic cleavage of the N-terminal arms of rE2/E3BP and rE2 over the experimental time period (∼ 3 days) required for the SE runs. However, negative-stain EM images (see the text below) of the samples prior to SE show complete cores; therefore, proteolytic cleavage during the course of the SE run is the more likely explanation. In our experience, full-length rE2/E3BP and rE2, particularly the flexible linker regions connecting the various domains, are highly susceptible to proteolysis over time. For example, gel-filtration chromatography (GFC) of 4-day-old rE2/E3BP resulted in the elution of several low-molecular-mass products (data not shown). This proteolytic cleavage is clearly seen on SDS-PAGE of post-SE rE2/E3BP and rE2 samples (Fig. 4a and b). rE2 and rE3BP migrate more slowly than expected on SDS-PAGE because they contain a large number of proline and alanine residues in their interdomain linkers that are thought to swell and induce bulkiness in the LDs.^45,46^ Apart from the expected monomeric rE2 (61 kDa) and rE3BP (50 kDa), SDS-PAGE reveals truncates with molecular masses of 50–60 kDa, 43 kDa (possibly SBD–CTD), 29 kDa (possibly CTD) and 20–25 kDa.

Global fitting of SE data yielded molecular masses of 5.71 and 4.59 MDa, respectively, for the minor species, lower than expected for a dimer of intact 60-meric rE2/E3BP (7.1 MDa) or rE2 (7.48 MDa) (data not shown) and not consistent with dimers of proteolytically cleaved cores. Moreover, the SE data could not be satisfactorily modelled with the self-association model in SEDPHAT, indicating that these minor species are aggregates rather than core dimers or other higher-order oligomers, consistent with the higher *s* tail observed in *c*(*s*) analysis of SV data (Fig. 3). Despite several attempts to minimise the extent of proteolytic degradation during purification by inclusion of protease inhibitors at all stages, the molecular masses of the cores were *always* in the range of 2.5–2.7 MDa (rE2/E3BP) and 3.0–3.1 MDa (rE2).

Analysis of the SE data for tE2/E3BP was carried out using a two-species model in SEDPHAT^43,44^ (Fig. 3c). Extrapolation of 1/*M*_app_ to infinite dilution for the predominant species (∼ 99% of total) gave *M*_w_^0^ = 1.65 ± 0.03 MDa (Fig. 3d), in good agreement with the value predicted from the tE2/E3BP amino acid sequence (1.67 MDa, based on the 48E2 + 12E3BP model), in support of the hypothesis that the human E2/E3BP core assembles via a ‘substitution’—and not ‘addition’—mechanism. Interestingly, because this truncated core lacks the N-terminal domains that are prone to proteolytic cleavage (as seen for rE2/E3BP and rE2), the experimentally determined molecular mass exactly matches that computed from the amino acid sequence. Global analysis and fitting for the minor species (∼ 1% of total) yielded a molecular mass of 2.29 MDa (data not shown), notably lower than that of the possible tE2/E3BP dimer (3.34 MDa, based on the 48E2 + 12E3BP model). In addition, fitting with the self-association model in SEDPHAT was unsatisfactory, suggesting the formation of minor aggregates rather than the presence of higher-order oligomers.

### SAXS and SANS measurements of rE2/E3BP, bE2/E3BP, and tE2/E3BP

**Fig. 5:**
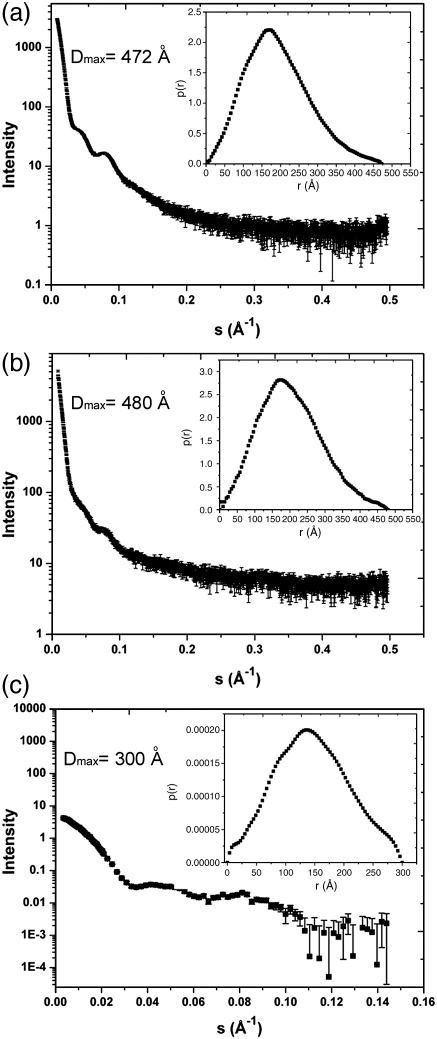
SAXS/SANS of rE2/E3BP, bE2/E3BP and tE2/E3BP. Scattering curves, together with distance distribution functions *p*(*r*) *versus r*, calculated using GNOM (insets) for (a) rE2/E3BP, (b) bE2/E3BP and (c) tE2/E3BP are shown. Error bars shown on the *p*(*r*) plots are not clearly visible owing to their small size.

SAXS data for rE2/E3BP and bE2/E3BP (native bovine PDC core) were acquired at various concentrations and temperatures (see Materials and Methods). The high-concentration samples aggregated, and the scattering curves from the low-concentration samples were dominated by noise. However, data obtained at concentrations of 830.9 nM (rE2/E3BP; Fig. 5a) and 183.1 nM (bE2/E3BP; Fig. 5b) were devoid of scattering from aggregates, interparticle interference effects, temperature-induced conformational changes or radiation-induced damage, and hence were used for ab initio modelling.

Guinier analysis and GNOM^47,48^ were employed to determine the radius of gyration *R*_g_ for rE2/E3BP (147 ± 1 and 148 ± 1 Å) and bE2/E3BP (158 ± 1 and 156 ± 1 Å). The distance distribution functions *p*(*r*) for rE2/E3BP and bE2/E3BP (Fig. 5a and b, insets) are generally bell shaped, but with slight deviations from the perfect Gaussian distribution (indicative of a spherical molecule), especially at high values of *r*. The maximum particle diameter *D*_max_ was determined to be 472 Å (rE2/E3BP) and 480 Å (bE2/E3BP), both much greater than the diameter of the native bovine heart and kidney E2/E3BP cores determined previously by cryo-EM (225 Å in the 5-fold orientation)^42^ probably because the extended flexible N-terminal domains have been imaged by SAXS, but not by this early cryo-EM study. The *R*_g_ (147 ± 1 Å) of rE2/E3BP is consistent with that determined by Hiromasa *et al.* (151 ± 2 Å), although the *D*_max_ they measured was lower (420 ± 10 Å).^37^

SANS curves were acquired for tE2/E3BP at three protein concentrations to account for interparticle interference effects. However, protein aggregation was observed for the highest sample concentration (712.6 nM), while data at the lowest concentration (161.7 nM) yielded insufficient signal-to-noise ratio. However, the scattering curve obtained at 353.3 nM showed no evidence of aggregates (Fig. 5c) and was used as data set for ab initio modelling. The *R*_g_ determined using Guinier analysis was 107 ± 2 Å. The *p*(*r*) distribution function is shown in Fig. 5c (inset). The *D*_max_ is 300 Å, and the *R*_g_ calculated from the *p*(*r*) function is 111 ± 2 Å, which agrees well with the value obtained from Guinier analysis.

### Low-resolution solution structures of human E2/E3BP cores

**Fig. 6:**
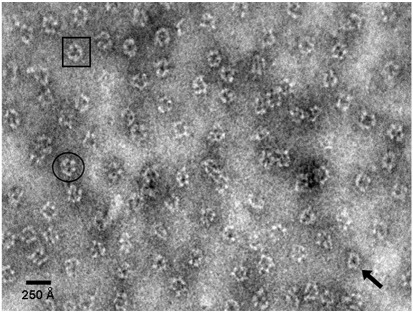
Solution structures of rE2/E3BP by negative-stain EM. Negative-stain EM of rE2/E3BP shows complete icosahedral cores with empty (pentagonal) faces. Orientation of cores along the 5-fold (arrow), 3-fold (square) and 2-fold (circle) axes is indicated.

Negative-stain EM of rE2/E3BP (Fig. 6) revealed a uniform distribution of well-formed icosahedral core structures with empty pentagonal faces, consistent with previous EM images for bovine heart PDC.^42^ Similar EM images of hollow cores with empty pentagonal faces were obtained for tE2/E3BP (data not shown). Core structures were clearly visible, exhibiting the underlying 5-fold, 3-fold and 2-fold structural symmetries (Fig. 6), although cores were more commonly imaged along the 5-fold axis, consistent with cryo-EM data for bE2/E3BP.^42^ This observation of empty pentagonal faces provides strong support for the ‘substitution model’ of the human E2/E3BP core assembly.

**Fig. 7:**
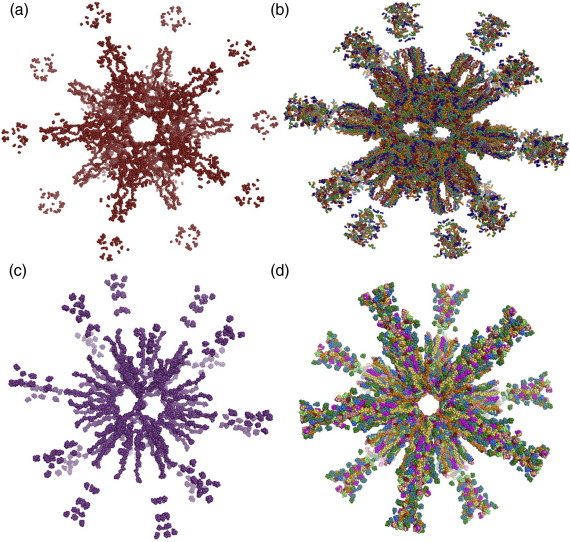
Solution structures of rE2/E3BP and bE2/E3BP by ab initio modeling from SAXS data. Single GASBOR reconstructions obtained from SAXS: (a) rE2/E3BP along the 5-fold axis of symmetry, and (c) bE2/E3BP along the 2-fold axis of symmetry. Ten single ab initio GASBOR reconstructions were superimposed to yield consensus models that preserved all key structural features of (b) rE2/E3BP (visualised along the 2-fold axis of symmetry) and (d) bE2/E3BP (visualised along the 5-fold axis of symmetry).

Ab initio models of rE2/E3BP (Fig. 7a) and bE2/E3BP (Fig. 7c) were generated from SAXS data with the program GASBOR^49,50^ by employing icosahedral symmetry and various penalty constraints during the modelling process (see Materials and Methods). Two hundred GASBOR runs were conducted to obtain consistent models (Fig. 7b and d). Several model families that satisfied the search volume were generated, but not all were consistent with structures observed with EM (i.e., dodecahedral structures with large hollow central cavities and empty pentagonal faces). Models similar to our own (Fig. 6) and published EM images^42^ were chosen for further analysis to obtain consensus models for both rE2/E3BP and bE2/E3BP. These consensus models were obtained by the superimposition of 10 ab initio GASBOR models for each of the cores (rE2/E3BP and bE2/E3BP; see Materials and Methods) in which the key structural features, such as the hollow cavities and extended peripheral arms, are preserved (Fig. 7). The positions of rE3BP within the cores cannot be ascertained from these models. Greater electron density is observed within the inner part of the core than in the peripheral flanking arms. This may be a consequence of the GASBOR algorithm favouring compactness. These limitations notwithstanding, the ab initio models clearly support the hypothesis that the human E2/E3BP core assembles via a ‘substitution’—and not ‘addition’—mechanism.

**Fig. 8:**
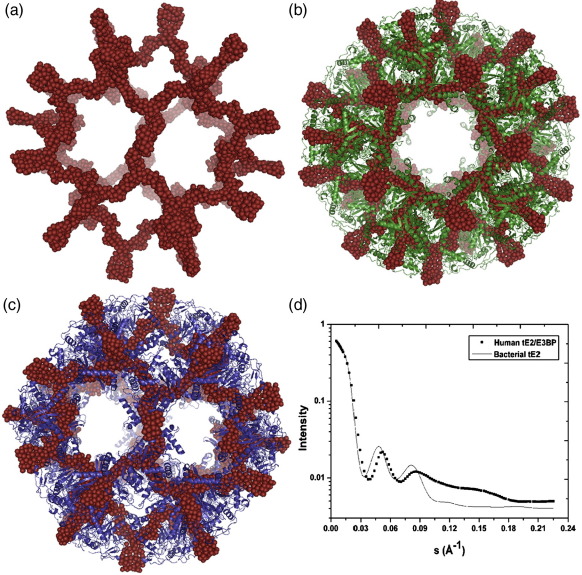
Solution structure of tE2/E3BP and its superimposition with the *B. stearothermophilus* tE2 crystal structure and a homology model of human tE2. The ab initio model of tE2/E3BP (generated from SANS data using GASBOR) is shown along the (a) 2-fold axis of symmetry. Superimposition of the ab initio model of tE2/E3BP (red) with (b) the crystal structure of *B. stearothermophilus* tE2 (green) and (c) the homology model of human tE2 (blue) indicates an overall conservation of icosahedral topology. (d) Overlay of the scattering curves of the ab initio SANS model of human tE2/E3BP and the crystal structure of bacterial tE2 using CRYSOL.

Ab initio restoration of a model for tE2/E3BP from SANS data using GASBOR^49,50^ produced an icosahedral core with hollow internal cavities and empty pentagonal faces (Fig. 8a). No density was observed within the pentagonal faces, as would occur if the CTD of E3BP were added to the inner or outer surface of the E2 core, as observed in yeast,^35^ thus strongly supporting the ‘substitution’ model of core organisation. Moreover, the solution structure of tE2/E3BP is consistent with the ‘inner core’ SAXS structures of full-length rE2/E3BP and bE2/E3BP (Fig. 7), as well as with the structures of bE2/E3BP^42^ and human tE2^32^ obtained from cryo-EM studies.^42^ Ten ab initio GASBOR models were successfully averaged using DAMAVER^51^ to obtain a consensus average structure of tE2/E3BP.

### Hydrodynamic and homology modeling of recombinant cores

Hydrodynamic bead models (HBMs) of rE2/E3BP and tE2/E3BP were generated from the ab initio models using the programs AtoB^52^ and TRANS2VORONOI (see Materials and Methods). Assuming a hydration of 0.4 g water/g protein, hydrated sedimentation coefficients of 28.3 S (rE2/E3BP) and 28.2 S (tE2/E3BP) were calculated using the program HYDRO^++^,^53,54^ in excellent agreement with the experimental values of 29.3 S (rE2/E3BP) and 27.5 S (tE2/E3BP) obtained from SV data. Additionally, the sedimentation coefficient of *B. stearothermophilus* tE2 was calculated for an HBM (generated from its atomic coordinates^28^ using AtoB^52^) using the program HYDRO^++^.^53,54^ On applying the hydration factor for 0.4 g water/g protein (0.87; see Materials and Methods), a sedimentation coefficient of 29.9 S was obtained for *B. stearothermophilus* tE2, slightly higher than the experimentally determined value of 27.5 S for tE2/E3BP. This difference is not likely to be the result of any difference in hydration, since the amino acid compositions of *B. stearothermophilus* tE2 and human tE2/E3BP are highly similar. Instead, it may indicate a more flexible open conformation for tE2/E3BP in solution, in comparison with the crystal structure for the bacterial truncate, stemming from so-called crystal packing effects. More importantly, it may also reflect slight differences in overall structures arising as a consequence of E3BP integration into the human E2 core.

In addition to AUC, SAXS and SANS, homology modelling was carried out to gain more insight into the human tE2 structure. The crystal structure of *B. stearothermophilus* tE2 (green; Fig. 8b) was used as template to generate the homology model of human tE2^28^ (blue; Fig. 8c). Superimposition of the ab initio SANS model of tE2/E3BP onto the crystal structure of *B. stearothermophilus* tE2^28^ (Fig. 8b) and the homology model of human tE2 (Fig. 8c) using SUPCOMB^55^ indicates good conservation of the gross structural features of the icosahedral core. The superimpositions indicate that despite variable regulatory functions and some minor structural differences (confined mainly to the hairpin domain and the N-terminal helix connected to the linker region preceding the CTD^32^), the overall icosahedral framework of the human E2/E3BP and *B. stearothermophilus* E2 CTD cores is highly conserved. Furthermore, intensity scattering curves calculated (using CRYSOL^56^) for the ab initio human tE2/E3BP model and the crystal structure of *B. stearothermophilus* tE2 are in good agreement with each other in the low-angle region of scattering that reflects overall molecular shape (Fig. 8d). The curves differ at wider angles because they originate from structures that are fundamentally different at this resolution (i.e., a crystal structure *versus* a dummy residue model).

### Cryo-EM reconstruction of cores

**Fig. 9:**
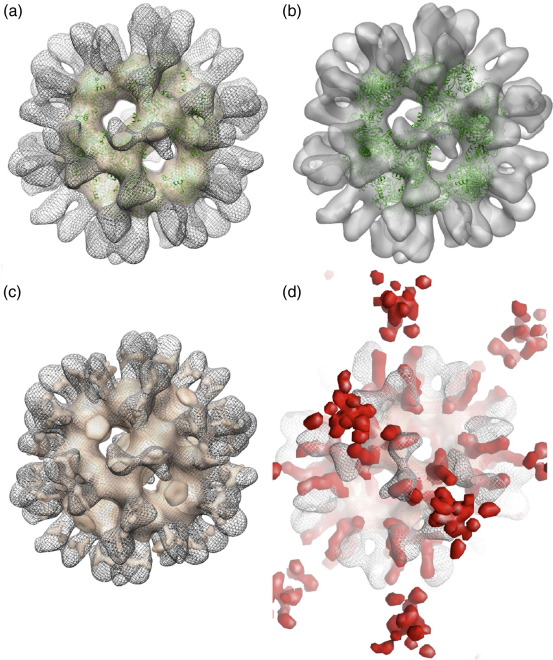
Cryo-EM of the full-length rE2/E3BP and rE2/E3BP:E3 complexes. (a) Cryo-EM reconstructions of the rE2/E3BP (semi-transparent surface) and E2/E3BP:E3 core (mesh), with the crystal structure of the *B. stearothermophilus* E2 core fitted within (green ribbon). The contour level of rE2/E3BP was chosen to match that of the complex with E3. (b) The E2/E3BP:E3 core only superimposed with the crystal structure of the *B. stearothermophilus* E2 core (green ribbon) for clarity. (c) The E2/E3BP core at a lower contour level showing the presence of satellite density, which represents relatively disordered regions of E2 and E3BP, superimposed on E2/E3BP:E3 in which these regions become ordered on E3 binding. (d) Close-up of the surface of the rE2/E3BP:E3 structure reported here (mesh) and the GASBOR-derived rE2/E3BP ab initio SAXS model (red). Extensions from the core surface found in SAS data agree with the positioning of the extensions that are relatively disordered without E3 and become ordered when it is present.

Images of rE2/E3BP and rE2/E3BP:E3 cores obtained under low-dose conditions using cryo-EM were subjected to three-dimensional reconstruction, as described in Materials and Methods. The refined maps show the well-defined dodecahedral geometry associated with PDC complexes, into which the crystal structure of the *B. stearothermophilus* E2 protein core fits well (Fig. 9a and b). When the density threshold for both rE2/E3BP and rE2/E3BP:E3 maps is set such that the core density superimposes, prominent and well-defined spikes remain extended from the latter structure, which are absent from the former (Fig. 9a). When the contour level of the rE2/E3BP map is lowered by ∼ 30%, additional density present beyond the core surface is revealed (Fig. 9c). Like the rE2/E3BP:E3 spikes, these densities are positioned on either side of the icosahedral 2-fold axis and align well with each other when the complexes are positioned within the common reference frame of their symmetry (Fig. 9c). This strongly suggests that the spikes in both maps derive, at least in part, from the same molecular components. It appears that the addition of E3 to the complex makes these extension regions more ordered, so that they are properly resolved on cryo-EM reconstruction. Furthermore, these spikes observed on the surface of the rE2/E3BP core at a lower contour level (Fig. 9c) are in the same positions as the linking density seen in the cryo-EM structure of the bovine kidney PDC core.^36^ We also compared the reconstructions to the SAXS-derived rE2/E3BP model computed ab initio using GASBOR. There is excellent agreement between the maps (Fig. 9d; see Materials and Methods for more details), and the cryo-EM data therefore strongly support the SAXS-derived model described above. The extensions from the core surface are longer in the SAXS model than in the cryo-EM models, but this derives from the different nature of the two techniques (see Discussion). Importantly, the binding of E3 to the E2/E3BP core results in increased density on the outside of the core icosahedron, in good agreement with earlier SAXS observations.^37^

### Assessment of core stability

The trimeric building blocks that make up the core of PDC serve as key elements for core stability.^28^ Cryo-EM studies of the yeast E2 core revealed variation in intertrimer distances, interpreted as ‘breathing’ of the core.^31^ Interestingly, integration of E3BP as an additional core subunit in eukaryotic PDCs may result in structural changes that mediate intertrimer distances and overall core stability. In this context, a comparative study of the core stabilities of rE2/E3BP, rE2 and tE2/E3BP was conducted using CD and fluorescence spectroscopy in the presence of the chemical denaturant GdmCl.

**Fig. 10:**
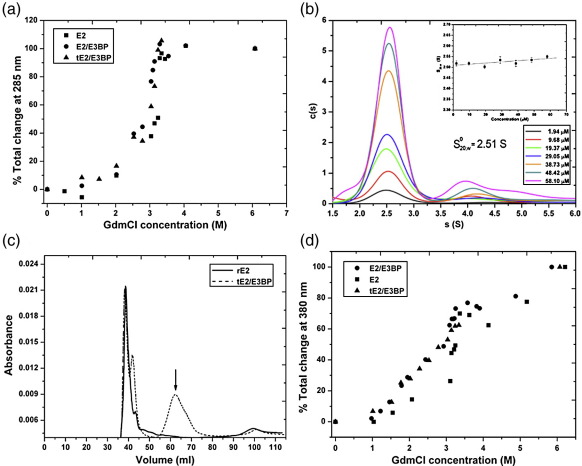
Recombinant core stability analysed via CD and fluorescence spectroscopy. (a) Comparative near-UV CD unfolding curves represented by the change in ellipticity at 285 nm in the presence of increasing quantities of GdmCl for all three cores (rE2/E3BP, rE2 and tE2/E3BP). (b) *c*(*s*) distribution derived from SV interference data for full-length rE3BP collected over a range of sample concentrations, along with (inset) determination of the standardised concentration-independent sedimentation coefficient for the main species (*s*_20,w_^0^ = 2.51 ± 0.02 S). (c) GFC of purified cores (tE2/E3BP and rE2) with 2.85 M GdmCl indicates differences in the elution profile. Elution peaks corresponding to void volume (⁎) and putative trimers of tE2/E3BP (arrow) are shown. (d) The percent change in unfolding monitored by tryptophan fluorescence spectroscopy as a function of GdmCl concentration indicates 50% of unfolded rE2/E3BP, rE2 and tE2/E3BP cores at 2.6, 3.1 and 1.75 M GdmCl, respectively. The wavelength (380 nm) was chosen as it showed a progressive trend from 0 to 6 M GdmCl.

Near-UV CD spectra of the cores in the presence of increasing concentrations of GdmCl resulted in loss of minima at 285 nm, with complete loss of structure observed by 6 M GdmCl. Unfolding/dissociation monitored via measurement of the total change in ellipticity at 285 nm indicated a high degree of structural perturbation of the cores in the near-UV region, with midpoints of unfolding being 2.70 M (rE2/E3BP), 2.88 M (tE2/E3BP) and 3.19 M (rE2) GdmCl (Fig. 10a). At concentrations less than 3 M GdmCl, a gradual unfolding/dissociation event is evident, as reflected in the progressive decrease in ellipticity. This is followed by an abrupt change beyond 3 M GdmCl that leads to complete unfolding/dissociation of the assembly.

The initial structural change (1.5–3 M GdmCl) may be attributed to the overall dissociation of the 60-meric cores to trimers, which then dissociate to monomers before complete unfolding (> 3 M GdmCl).^57^ The peak at 285 nm corresponds to spectral contribution from aromatic amino acid residues (such as tryptophan and tyrosine) and is rapidly lost with increasing GdmCl concentration. This may be attributed to conformational changes in the environment of aromatic residues, resulting in alterations to the overall tertiary structure during unfolding/dissociation. In addition, sigmoidal curves (Fig. 10a) suggest unfolding via some intermediates, consistent with previously published refolding studies on bE2/E3BP.^57^ Additionally, the Gibbs free energy of unfolding Δ*G* of the cores was determined to be 17.0 kJ/mol (rE2/E3BP), 23.6 kJ/mol (rE2) and 11.0 kJ/mol (tE2/E3BP) (data not shown), further corroborating the unfolding trend as observed from the near-UV CD data. In summary, stability curves obtained from near-UV CD indicate that rE2 is more stable than tE2/E3BP, which in turn is more stable than rE2/E3BP, suggesting destabilisation of the core upon integration of E3BP.

This is further supported by SV AUC data for full-length E3BP (rE3BP) indicating a predominant monomeric species (*s*_20,w_^0^ = 2.51 ± 0.02 S) and a small fraction of possible dimer (Fig. 10b). SE data for rE3BP could be well fitted by a two-species model with a major species of *M*_w_^0^ = 49.6 kDa, in good agreement with the molecular mass calculated from the amino acid sequence (51,636 Da), and by a minor species with a molecular mass lower than that of an rE3BP dimer (data not shown). The SE data could not be satisfactorily fitted with self-association models in SEDPHAT, suggesting the formation of minor irreversible aggregates rather than higher oligomers. In summary, the AUC data show conclusively that rE3BP, unlike rE2, does not spontaneously self-associate to form 60-meric cores, but in fact remains largely monomeric in solution. This is consistent with a role for rE3BP in destabilising rE2/E3BP complexes, since any trimer–trimer interaction that involves contact between rE3BP molecules is likely to be much weaker than the strong rE2–rE2 interactions that are key to the spontaneous formation of rE2. These findings lend strong support to the hypothesis that E3BP destabilises the E2/E3BP core assembly.

The difference in core stability between rE2 and tE2/E3BP was additionally confirmed by gel filtration in the presence of 2.85 M GdmCl, close to the midpoint of unfolding for tE2/E3BP (2.88 M) but relatively far from that for rE2 (3.19 M). Gel-filtration profiles indicate void volume elution (40 ml) of the intact 60-meric cores of both tE2/E3BP and rE2 (Fig. 10c). However, an additional peak at 62 ml is observed only with tE2/E3BP. Previous refolding studies by McCartney *et al.* showed the possible formation of trimers and monomers during the unfolding of bE2/E3BP.^57^ The apparent molecular mass of the additional tE2/E3BP peak is 185 kDa, consistent with the presence of E2 homotrimers (183 kDa) and/or E2/E3BP heterotrimers (2E2 + 1E3BP; 174 kDa). It is interesting to note that, at 2.85 M GdmCl, while the gel-filtered tE2/E3BP reveals subunit dissociation into trimers, rE2 remains almost intact (Fig. 10c), consistent with the greater stability of the E2 oligomeric assembly.

Fluorescence spectra were recorded by monitoring the intrinsic fluorescence of tryptophans in rE2/E3BP, rE2 and tE2/E3BP. In rE2/E3BP, there are four tryptophans in the rE2 subunit (one in outer LD, one in inner LD and two in CTD) and three in the rE3BP subunit (one in LD, one in inner linker and one in CTD). Similarly, in tE2/E3BP, there are two tryptophans in tE2 and one tryptophan in tE3BP. A gradual shift of the peak maximum towards higher wavelengths with increasing concentrations of GdmCl was observed for all cores. In the fully folded native state, the peak is at 328–331 nm, indicative of tryptophans buried in the core. Increasing amounts of GdmCl drove a gradual redshift beginning at 1.5 M (rE2/E3BP) and 1.75 M (tE2/E3BP) GdmCl and resulting in a shift to 334 nm, suggesting local dissociation and partial solvent exposure of tryptophans. On increasing the concentration of GdmCl further, a dramatic shift of the maximum emission peak to 352 nm was observed at 2.5 M (rE2/E3BP and tE2/E3BP) and 3 M (rE2) GdmCl, progressing to 360 nm by 6 M GdmCl, suggesting complete solvent exposure of all tryptophans brought about by the overall unfolding/dissociation of the cores (data not shown).

In summary, the early changes in the observed tryptophan fluorescence between 0 and 2 M GdmCl, indicative of local perturbation in structure and/or formation of trimeric intermediates, are similar to previous studies of bE2/E3BP demonstrating the formation of trimeric intermediates between 1.8 and 2 M GdmCl.^57^ This is immediately followed by a phase of rapid denaturation, with about 50% unfolded/dissociated core observed by 1.75 M (tE2/E3BP), 2.6 M (rE2/E3BP) or 3 M (rE2) GdmCl (Fig. 10d), corresponding to an overall unfolding event with a major loss of quaternary and secondary structures. This trend in unfolding observed by fluorescence correlates well with that observed in the near-UV CD data (Fig. 10a). As fluorescence emission is a combination of signals from all tryptophans, it is not possible to draw conclusions about detailed structural changes in rE2/E3BP and rE2. However, as LDs and SBDs are missing from tE2/E3BP, it is possible to conclude that in the range of GdmCl corresponding to the dissociation of the overall quaternary structure into trimeric intermediates, the change in emission wavelength is accounted for solely by changes in the environment of the inner CTD tryptophans.

It is also interesting to note that the intensity changes for the full-length (rE2/E3BP and rE2) cores differ from those for tE2/E3BP. An initial decrease in fluorescence intensity at low GdmCl concentrations, followed by a gradual increase primarily associated with the exposure of N-terminal domain tryptophans to solvent, was observed for full-length cores (data not shown). This is likely to contribute greatly to the observed redshift. In contrast, the steady increase in fluorescence with increasing GdmCl concentrations for tE2/E3BP was probably due to the gradual exposure of the CTD tryptophans that may be largely quenched in the folded state (data not shown).

## Discussion

### New insights into the basic architecture and general properties of E2/E3BP core assembly

Although recombinant human and yeast truncated E2 core structures have been determined previously by cryo-EM,^29,32,35^ there is no corresponding structure for the functional human E2/E3BP core (or any full-length E2/E3BP core) to date. Recombinant human or native bovine E2/E3BP cores have resisted crystallisation, owing primarily to the intrinsic flexibility of their N-terminal flanking regions and the difficulties in obtaining high yields of truncated E2/E3BP; consequently, our understanding of the structure–function relationships in the human PDC core has remained limited. Attempts to obtain truncated E2/E3BP have always employed limited proteolysis of the core with trypsin.^58^ Yu *et al.* have been successful in producing only minimal yields of recombinant human truncated E2 for structural studies.^32^ In contrast, high yields (5–8 mg/l bacterial culture) of pure recombinant full-length E2, E2/E3BP and tE2/E3BP were successfully obtained in this study and used for structural characterisation.

Our SAXS and SANS models represent the first solution structures for full-length recombinant and native bE2/E3BP. Native bovine and recombinant human assemblies are indistinguishable from each other at this resolution, indicating that the intact 60-meric recombinant human E2/E3BP core, with its characteristic pentagonal dodecahedral framework, can assemble successfully in *Escherichia coli*. The existence of a characteristic 60-meric core formed from a combination of E2 and E3BP subunits is in agreement with an early EM reconstruction for bovine PDC;^42^ moreover, superimposition of the tE2/E3BP SAXS model on the crystal structure for truncated *B. stearothermophilus* E2 reveals that the overall dimensions and basic underlying morphology are conserved in the mixed E2/E3BP subunit core assembly.

Interestingly, both full-length bovine and human E2/E3BP structures feature elongated ‘arms’ emanating outwards from an icosahedral inner core with empty pentagonal faces. These protruding ‘arms’ account for approximately 40% of the overall *D*_max_ (480 Å) for rE2/E3BP (as opposed to 300 Å for tE2/E3BP). They are also notably absent from the corresponding tE2/E3BP SAXS model and thus represent the first direct visualisation of the N-terminal flexible LDs and SBDs of E2 and E3BP within the context of an intact, fully assembled ‘core’ structure. In addition, the presence of open (unoccupied) pentagonal faces is a striking common feature of all three core reconstructions, lending further strong support to the ‘substitution’ model of subunit organisation, and in direct contrast to the earlier ‘addition’ model proposed for yeast E2/E3BP.^35^ The overall dodecahedral morphology with empty pentagonal faces was further confirmed by negative staining and cryo-EM of the rE2/E3BP assembly.

This study also reports on novel features of the first cryo-EM images of rE2/E3BP on its own, as well as complexed with E3. In addition to confirming the presence of unoccupied pentagonal faces, these reconstructions resolve density deriving from the regions of E2 and E3BP extending above the main core, which are absent from published crystal structures. The location of these extensions agrees extremely well with the positioning of the linking density previously reported for cryo-EM of the bovine kidney PDC core,^36^ as well as with the equivalent regions of the ab initio GASBOR SAXS model of rE2/E3BP, reinforcing the validity of this structure. Furthermore, addition of E3 renders these extensions much more ordered, as evidenced by the appearance of well-defined spikes, although they are not resolvable to the same distance from the core surface as envisaged via SAXS, reflecting basic differences in the structural detail provided by cryo-EM and small-angle scattering (SAS) techniques (see the text below). Previous quasi-elastic light scattering and cryo-EM studies of the bovine heart and kidney PDC also established that E2/E3BP cores have an icosahedral framework with pentagonal faces^42^ and a maximum dimension of ≈ 400 Å.^59^ However, the anticipated high level of flexibility of the outer N-terminal domains precluded their resolution in EM micrographs. Additionally, only the maximum dimensions of bovine heart PDC core and its associated E2/E3BP core were obtained from the quasi-elastic light scattering studies.

Our complementary SE analyses also lend strong support to the substitution model of core organisation, as the observed molecular mass of rE2/E3BP is lower than that of rE2, consistent with earlier observations by Hiromasa *et al.*^37^ Substitution of E2 by E3BP in the 60-meric core reduces the overall mass because the E2 polypeptide has an *M*_r_ lower than that of E3BP. Both rE2 and rE2/E3BP have high frictional ratios (*f*/*f*_0_) and large hydrodynamic radii (*R*_s_), largely due to the solvent-exposed elongated outer linkers and LDs of E2 and E3BP and the presence of open hollow faces.^59^ Interestingly, it is observed that *f*/*f*_0_ and *R*_s_ for rE2 in our study are higher than those for rE2/E3BP, in direct contrast with previously reported AUC data for these cores.^37^ This is likely to be a consequence of the highly anionic nature of the N-terminal domains of E2 (LDs and linkers have a total charge of − 14), the large number of amino acids (> 120) in the flexible linker regions of E2 that connect the various domains, and the high alanine and proline contents of these linkers (Fig. 1).

Our current data also provide the first evidence relating to the oligomeric state and capacity for self-assembly of recombinant full-length E3BP produced as an independent species, since *in vivo*, this polypeptide exists exclusively in association with E2 as an integral subunit of the core assembly. Interestingly, despite its significant homology (∼ 50%) and similar domain organisation to E2, E3BP is largely monomeric in solution, exhibiting only a weak tendency for irreversible aggregation. These experimental data support a prediction, based on an analysis of sequence alignments and bacterial E2 crystal structures, that E3BP will lack key residues/motifs involved in self-assembly and the hydrophobic ‘ball-and-socket’ joints responsible for intertrimer interactions. These E2-mediated contacts are vital to the integrity and stability of the 60-meric pentagonal dodecahedron assembled from the coalescence of 20 basic trimeric units. However, in contrast to extensive interdigitated associations among the E2 monomers within trimeric units, the contacts between adjacent trimers are very limited.^28^ The presence of E3BP as an integral component of trimeric units (as predicted by the ‘substitution model’) should further weaken intertrimer interactions, since E3BP lacks the key residues involved in mediating trimer–trimer contacts.^37^

Our current data from AUC (for full-length rE3BP), CD and fluorescence spectroscopy studies (on full-length and truncated E2/E3BP and full-length E2) provide the first direct evidence in support of the above hypothesis. Near-UV CD spectroscopy in the presence of GdmCl clearly shows unfolding via intermediates, in good agreement with previous unfolding studies of the bE2/E3BP^57^ demonstrating initial dissociation into putative trimers and strongly suggesting that the introduction of E3BP into the E2 core has a significant effect on its stability. Thus, lower GdmCl levels were required to induce disruption of intact rE2/E3BP and tE2/E3BP compared with rE2. The higher stability of rE2 (compared with rE2/E3BP or tE2/E3BP) is clearly reflected in their midpoints of unfolding, as well as in an analysis of trimer formation by GFC, further supporting the hypothesis of decreased E2/E3BP core stability. Moreover, early changes in core fluorescence (at low GdmCl concentrations) prior to large-scale disruption of secondary structure reflect the partial dissociation of the 60-meric core leading to the release of putative trimers.

Current core E2/E3BP models also highlight the presence of large internal solvent-filled cavities, which are characteristic features of these eukaryotic assemblies. The elevated *D*_max_ (300 Å) of tE2/E3BP obtained by SANS, compared with that previously observed for tE2 cores (225–272 Å),^31,42,60^ may reflect a greater size heterogeneity or extent of ‘breathing’ in E2/E3BP. As the concept of breathing has been studied only with the yeast E2 core,^31^ it is unclear how it modulates the size, flexibility and function of human E2/E3BP, and, more specifically, how the introduction of E3BP affects these processes. It has been speculated that breathing of the core enhances the movement of the lipoyl ‘swinging arms’ towards the catalytic centres, while additionally augmenting substrate channeling. In addition, the new structures of the E2/E3BP cores presented here suggest that access to the active sites by the LDs will be further boosted by the open topology of the empty faces. These architectural features are suggested to enhance the overall rates of catalysis.^31^

### Comparison of cryo-EM and SAXS images: Technical considerations

Cryo-EM images are inherently noisy, and signal is recovered by assigning relative orientations to objects and combining them into single three-dimensional volumes. This averaging smears out any features not consistently positioned on the images. Application of symmetry constraints smears out anything not following the symmetry, whether due to disorder *or* partial occupancy. If partial occupancy *is* the reason, then the shape of the object is consistently present, but its level of density compared with the rest of the structure is reduced because it is averaged against empty binding sites. Thus, use of a lowered contour level can allow the recovery of an impression of the partially occupied structure. The need for a lower contour level to recover the density observed when E3 binds to the core (Fig. 9c) is consistent with partial occupancy of the sites to which E3 binds, in comparison with the total number of projections recovered. The volume occupied by this density is consistent with the volume of an E3 dimer plus an associated dimer of the E3BP SBD and linker; however, interestingly, the atomic resolution structure of the E3 dimer complexed with E3BP SBD^61,62^ does not satisfactorily superimpose with the density. This suggests that the interaction observed between E3 monomers in the crystal may not reflect the interactions on the PDC core surface. This disagreement could stem from the presence of E3 cross-bridges linking pairs of E3BP dimers on the core surface.

Cryo-EM maps that suffer from partial occupancy/disorder will display noisy features that cannot be distinguished rigorously from literal noise in the background of the image as the contour is lowered. These are therefore typically ignored. However, solution SAS data represent a spherical average of the sample object. These are modelled as a set of discrete ‘atoms’, in the case of PDC, according to symmetry. In this method of volume reconstruction, the statistical presence of a feature separated from the main body of the structure (which appears as noise in a cryo-EM map) is referenced by the presence of an ‘atom’. Since the ‘atoms’ are discrete objects, they allow such features to be represented separately from background noise, which is not possible with cryo-EM. Indeed, we observed that further lowering the contour level of the cryo-EM reconstructions (Fig. 9c) does reveal density extending far from the core surface, as the SAXS model does and as previously noted^63^ (data not shown), but we prefer to concentrate on the portions of the structure that we can characterise with confidence, and these agree with the regions of the extension structures near the core surface in the scattering model.

An additional feature is that in the imaging of an object by cryo-EM, no *a priori* decision can be taken as to what is signal and what is noise, and the image formation process inevitably suffers from effects of inelastic scatter, sample movement and inaccurate correction of contrast transfer function (CTF). There is no CTF in SAS: this is the reason that SAS data can be used to correct the amplitude component of the CTF. The assumption of spherically averaged data in SAS aids in signal-to-noise ratio; however, in cryo-EM, the views obtained are considered to be specifically defined orientations and cannot be averaged with other views without an orientation assignment being made, which is itself subject to artifacts associated with noise. Taken together, these findings strongly support the hypothesis that cryo-EM imaging misses key features that can be detected by SAXS studies.

## Concluding Remarks

In summary, this work provides important new insights into the structural organisation and general features of the E2/E3BP core assembly of mammalian PDC (a) by an analysis of several recombinantly produced and native core variants and (b) by a comparison of the SAXS/SANS and cryo-EM structures for these assemblies. These models all display a common pentagonal dodecahedral framework with open pentagonal faces that are, at the resolution attained, indistinguishable in overall geometry and dimensions from the crystal structure for the E2-only core of *B. stearothermophilus*. They also provide definitive evidence in support of the ‘substitution model’ of core organisation in which E3BPs replace an equivalent number of E2s within this 60-meric assembly rather than being located on its 12 pentagonal faces, as envisaged in the earlier ‘addition model’. However, these low-resolution structures still do not permit E2 subunits to be distinguished from their E3BP counterparts and thus are unable to provide information on the overall subunit composition or precise locations of E3BPs within the core.

An advantage of this approach, however, is that it has permitted the first direct visualisation of the peripherally extended N-terminal arms of E2 and E3BP encompassing their LDs and SBDs, joined by flexible linkers in the context of an intact core assembly. In addition, by comparison with cryo-EM images of the E2/E3BP core in the presence and in the absence of bound E3, it is possible to identify those regions immediately above the core surface housing the SBDs. Moreover, these EM reconstructions have revealed that E3 induces striking changes in this region, as evidenced by the appearance of well-defined spikes. These indicate the formation of more ordered structures that are compatible with the existence of a network of E3 cross-bridges linking pairs of E3BPs around the core surface, as proposed previously by our group.^69^ Finally, the ability to produce rE3BP both as an independent polypeptide and in recombinant E2/E3BP cores has allowed assessment of its oligomeric state, its inherent capacity for self-association and monitoring of its effects on core assembly and stability. In contrast to E2, E3BP shows no capacity for reversible self-association and exists largely as a monomeric species in solution. In line with this observation, inclusion of E3BP within the core assembly appears to decrease overall core stability. However, its contribution to mediating core dynamics, the overall rates of catalysis, the phenomenon of ‘breathing’ and the observed size heterogeneity of the mammalian core assembly remain to be established.

An urgent priority is to distinguish between the current 48 + 12 model and the current 40 + 20 model for the E2BP core assembly, requiring determination of the number and distribution of E3BPs within the mammalian core structure. However, this is a challenging task and requires novel approaches to the problem. Single-molecule studies and/or cryo-EM of the E2/E3BP core complexed with a monoclonal antibody to E3BP offers hope for this in the near future. In addition to validating the subunit composition of the E2/E3BP core, these techniques should also provide more detailed information on the molecular basis of core heterogeneity, size variation and intrinsic protein dynamics.

## Materials and Methods

### Cloning of tE2/E3BP

The C-terminal constructs of mature E2 and E3BP (tE2 and tE3BP) typically encompassed the C-terminal region and several additional residues of the preceding linker region. Primers were obtained from MWG Biotech (UK) to enable isolation of the C-terminal clones tE2 (aa 398–613) and tE3BP (aa 245–501). While tE2 was cloned into the NdeI and BamHI sites of vector pET11b via the TOPO/TA Cloning System (Invitrogen), tE3BP was cloned directly into pET28b using the restriction sites BamHI and XhoI via the classical cloning approach. All PCR products and purified DNA were analysed on a 1% (wt/vol) agarose gel. Successful cloning of inserts was confirmed by diagnostic digests and DNA sequencing of recombinant plasmids.

### Expression and purification of rE2/E3BP, rE2, and tE2/E3BP

The recombinant cores rE2 (in pET14b), rE2/E3BP and tE2/E3BP (in pET11/pET28b) and full-length E3BP (rE3BP) were overexpressed in the *E. coli* strain BL21 star (DE3) (Invitrogen) and grown in LB media (500 ml) containing 100 μg/ml ampicillin (rE2, rE2/E3BP and tE2/E3BP) and 25 μg/ml kanamycin (rE2/E3BP, tE2/E3BP and rE3BP). Bacteria were grown at 37 °C to an OD_600_ of 0.6–0.8 and subsequently induced with 1 mM IPTG for 17 h at 18 °C (rE2) and 15 °C (rE2/E3BP, tE2/E3BP and rE3BP). On induction, the rE2, rE2/E3BP and rE3BP cultures were further supplemented with 50 μg/ml lipoic acid. Cells were then harvested by centrifugation at 10,000***g*** for 15 min at 4 °C, and overexpression was analysed by SDS-PAGE. Frozen cell pellets were resuspended in 20 ml of metal chelate binding buffer [100 mM NaCl, 10 mM imidazole, and 50 mM KH_2_PO_4_ (pH 8.0)] supplemented with Complete EDTA-Free Protease Inhibitor Tablets (Roche), DNase I (Sigma) and Halt Protease Inhibitor Cocktail (10 μl/ml binding buffer; Thermo Scientific). Cells were lysed in a French pressure cell at 950 psi and subsequently centrifuged at 10,000***g*** for 15 min at 4 °C. The soluble supernatant was removed, filtered through a 0.2-μm syringe filter and injected onto a zinc metal chelate affinity column (20MC) on the BioCAD Sprint or BioCAD 700E chromatography workstations (Applied Biosystems, USA). While the N-terminal His tag is present in rE2 and rE3BP, it is located only in E3BP in the rE2/E3BP and tE2/E3BP complexes. Proteins were eluted as 1.5-ml fractions with a 0–100% imidazole gradient of elution buffer [100 mM NaCl, 500 mM imidazole and 50 mM KH_2_PO_4_ (pH 6.0)], and yield and purity were analysed by SDS-PAGE. Protein fractions were then pooled and subjected to either anion-exchange chromatography (rE2, rE2/E3BP) or gel filtration (tE2/E3BP and rE3BP) for further purification.

Pooled fractions of rE2 and rE2/E3BP from the metal chelate column were exchanged into dialysis buffer [2 mM ethylenediaminetetraacetic acid (EDTA), 450 mM NaCl and 25 mM Tris–HCl (pH 7.5)] and subjected to 20HQ anion-exchange chromatography (Applied Biosystems) for selective removal of DNA. The column was equilibrated with binding buffer [2 mM EDTA and 25 mM Tris–HCl (pH 7.5)], and protein was eluted via a 0–100% gradient of elution buffer [2 mM EDTA, 2 M NaCl and 25 mM Tris–HCl (pH 7.5)]. Protein obtained from anion-exchange chromatography (rE2 and rE2/E3BP) or metal chelate chromatography (tE2/E3BP and rE3BP) was pooled, concentrated and further purified by GFC on a Sephacryl S-300 column (Amersham, USA) equilibrated with PEBS100 buffer [2 mM EDTA, 0.01% (wt/vol) NaN_3_, 100 mM NaCl and 50 mM KH_2_PO_4_ (pH 7.5)]. Absorbance was monitored at 260 and 280 nm, and protein fractions (2 ml/tube) were analysed by SDS-PAGE. The concentration of the purified cores was measured at 280 nm on an Ultrospec 4300 Pro UV/Vis spectrophotometer. The extinction coefficients of the cores calculated from protein sequences via PROTPARAM† were 20,970 M^−^ ^1^ cm^−^ ^1^ (rE3BP), 2,124,600 M^−^ ^1^ cm^−^ ^1^ (rE2), 1,951,320 M^−^ ^1^ cm^−^ ^1^ (rE2/E3BP) and 862,200 M^−^ ^1^ cm^−^ ^1^ (tE2/E3BP) based on the 48E2 + 12E3BP model.

### Purification of bovine heart PDC core and bovine E2/E3BP core

PDC was purified from bovine heart essentially as described before^64^ and involved biasing the maximum yield of PDC (without any OGDC contamination) by a final precipitation step adding 0.06 vol of 35% (wt/vol) PEG. PDC concentration was determined using the Bradford assay and was stored in small aliquots (at 10 mg/ml) in 50% (vol/vol) glycerol at − 20 °C for future use. Bovine E2/E3BP core (bE2/E3BP) was extracted from PDC by discontinuous sucrose gradients as described earlier,^58^ with the following changes: 4 ml of 20% (wt/vol) sucrose, 2 ml of 10% (wt/vol) sucrose and 2 ml of 5% (wt/vol) sucrose in PEBS2M buffer [2 M NaCl, 2 mM EDTA, 0.01% (wt/vol) NaN_3_, 1% (vol/vol) Triton X-100 and 50 mM KH_2_PO_4_ (pH 7.4)] were layered one above the other. Bovine heart PDC (5–8 ml) was layered on the sucrose gradients and ultracentrifuged in a Beckman Ti70 rotor at 182,000***g*** at 4 °C for 150 min. Supernatant fractions (1 ml) were collected from the top using a peristaltic pump, and the bE2/E3BP pellet was suspended in storage buffer as mentioned above. The protein concentration of bE2/E3BP was measured using the Biuret method at 550 nm.

### Analytical ultracentrifugation

SV experiments were performed on a Beckman Coulter Optima XL-I analytical ultracentrifuge (Beckman Coulter, Palo Alto, CA, USA) using an An-50 Ti eight-hole rotor. Samples (360 μl) at concentrations from 76 to 306 nM (rE2/E3BP), 60 to 260 nM (rE2), 116 to 730 nM (tE2/E3BP) and 1.94 to 58.1 μM (rE3BP), along with PEBS100 buffer [2 mM EDTA, 0.01% (wt/vol) NaN_3_, 100 mM NaCl and 50 mM KH_2_PO_4_ (pH 7.5)] as reference solvent, were loaded into 12-mm path-length charcoal-filled epon-double-sector centrepieces and spun at 20,000 rpm (rE2, rE2/E3BP and tE2/E3BP) and 45,000 rpm (rE3BP) at 4 °C, and a series of scans was collected using either interference optics, absorbance optics or a combination of both. Data were recorded over a radial range of 6.0–7.25 cm, and a radial step size of 0.002 cm was used in the case of absorbance optics. For interference optics, 450 scans were recorded 1 min apart, and laser delay was adjusted prior to the run to obtain high-quality interference fringes. Data were analysed using the program SEDFIT.^65,66^ Sedimentation boundaries were initially modelled as numerical finite-element solutions of the Lamm equation using the *c*(*s*) analysis. Apparent sedimentation coefficients were further obtained via the non-interacting discrete species model that employs finite-element analysis. The apparent sedimentation coefficients were then corrected to standard conditions of temperature and solvent before being extrapolated to infinite dilution to obtain a sedimentation coefficient independent of concentration, *s*_20,w_^0^. The partial specific volume *v̄* of rE2/E3BP and rE2 calculated from their amino acid compositions using the program SEDNTERP‡^67^ was 0.744 ml/g at 20 °C, while those of tE2/E3BP and rE3BP were calculated to be 0.746 and 0.742 ml/g, respectively, at 20 °C. As both SV absorbance and interference data yielded the same results, only interference data have been presented in this work, unless otherwise stated.

SE experiments were conducted in a Beckman Coulter Optima XL-I analytical ultracentrifuge using an An-50 Ti eight-hole rotor at speeds of 3000, 5000 and 7000 rpm. However, analysis of the data at 5000 and 7000 rpm was unsatisfactory, yielding very steep exponential solute distributions. Hence, all subsequent SE studies were performed at 3000 rpm, unless otherwise stated. All experiments were carried out at 4 °C, with protein samples (80 μl) at various concentrations from 154 to 461 nM (rE2/E3BP), 60 to 260 nM (rE2) and 251 to 726 nM (tE2/E3BP) loaded into 12-mm path-length charcoal-filled epon-double-sector centrepieces. PEBS100 buffer was used as reference solvent. After an initial delay of 20–24 h, a series of scans (12–15) separated by 3 h was recorded using interference optics. Data were recorded over a radial range of 6.8–7.25 cm, with the laser delay adjusted before the run. The program WinMATCH§ was used to confirm that the system had reached equilibrium, and the SE data were analysed using the program SEDPHAT.^43,44^ Single data-set analysis was performed for every concentration to obtain an apparent whole-cell weight-average molecular mass, *M*_app_. The average molecular mass independent of concentration was determined by plotting 1/*M*_app_ *versus* concentration, with the *y*-intercept yielding the whole-cell average molecular mass at infinite dilution *M*_w_^0^.

### Small-angle X-ray scattering

SAXS experiments were performed on beamline X33 of the European Molecular Biology Laboratory (EMBL)/Deutsches Elektronen Synchrotron (Hamburg, Germany). Data were collected in mica sample holders at various sample concentrations (rE2/E3BP: 140.8, 830.9 and 1070.4 nM; bE2/E3BP: 183.1 and 321.1 nM) and temperatures (10, 20 and 37 °C) at a detector distance of 4 m over a momentum transfer range of 0.008 < *s* < 0.497 Å^−^ ^1^ (where *s* = 4πsinθ/λ). The 345-mm two-dimensional MAR image plate detector was calibrated using bovine serum albumin as standard prior to the experiment. Each data set was recorded over a period of 4 min, with data for buffer (PEBS100) collected before each sample. Scattering data were integrated, normalised to the main incident beam and detector response, and processed using the program PRIMUS‖.^68^ Scattering curves were assessed for radiation damage, and those unaffected by aggregation were then averaged, buffer subtracted and scaled for concentration using PRIMUS. The final average buffer-subtracted curve was inputted to GNOM,^47,48^ and the *p*(*r*) distance distribution plots and maximum dimension *D*_max_ of the proteins were determined. The radius of gyration *R*_g_ was obtained from Guinier analysis (in PRIMUS) and GNOM.

### Small-angle neutron scattering

SANS was conducted on beamline D22 of the Institut Laue Langevin (ILL; Grenoble, France). Samples were measured in protein buffer (PEBS100) at detector distances of 4 and 14 m, covering an overall *Q* range of 0.0034 < *Q* < 0.143 Å^−^ ^1^ (where *Q* = 4πsinθ/λ). Scattering data were recorded at 4 °C in 1-mm path-length quartz cuvettes at protein concentrations of 1039.4 nM for rE2/E3BP and 161.7, 353.3 and 712.6 nM for tE2/E3BP. Transmission and scattering data for buffer and sample at both detector distances were collected for 4 and 15 min, respectively. The response of the two-dimensional area gas detector was calibrated by measuring the scattering of H_2_O. While the Unix program MAD was used to control data acquisition, the GUI program GRAS_ans_P written by Charles Dewhurst (ILL)¶ was employed for graphical inspection, analysis and reduction of raw data. The *p*(*r*) distance distribution, maximum dimension *D*_max_ and radius of gyration *R*_g_ were obtained using the programs PRIMUS and GNOM, as described above.

### Ab initio modelling of SAXS and SANS data

All ab initio reconstructions of molecular envelopes from SAXS and SANS data were generated using the program GASBOR.^49,50^ Icosahedral (PICO) symmetry was applied with no constraint on disconnectivity and peripheral penalties during the modelling process. As GASBOR runs were extremely time intensive (10 days on a desktop PC) due to the large size of the cores, 100–200 simulations/protein were conducted in batch mode using the powerful computer grid system SCOTGRID^a^. Each simulation on SCOTGRID took approximately 7 days to complete on a single computer node. GASBOR models of tE2/E3BP were superimposed and averaged by the program DAMAVER^51^ to obtain a consensus average structure. However, averaging 10 ab initio models of rE2/E3BP and bE2/E3BP to obtain consensus structures posed significant problems on account of hollow cavities within the cores and peripheral regions of elongated density (from SBDs and LDs) within the same structure. Nevertheless, as volumes obtained from CRYSOL^56^ (4.3–4.37 × 10^6^ Å^3^) for each reconstruction compared favourably with the calculated volumes of the cores (4.38 × 10^6^ Å^3^), representing consensus models of rE2/E3BP and bE2/E3BP by the superimposition of 10 ab initio GASBOR models proved to be a feasible approach to overcoming the problem of structure restoration encountered with DAMAVER.

### Negative-stain EM

Negative-stain EM was performed on a 1200 EX scanning microscope (JEOL, Japan) at an operating magnification of 30,000× and with an acceleration voltage of 120 kV. Carbon-coated copper grids (400 mesh; Agar Scientific Ltd., UK) were initially rendered hydrophilic by plasma glow discharge, after which 5 μl of protein was added and loaded onto each grid. The grids were then sequentially washed with distilled water (50-μl droplets) and, finally, with a 50-μl droplet of 2% (wt/vol) ammonium molybdate (pH 7.2) (negative stain; Agar Scientific Ltd.). Excess liquid was drained from the grids using Whatman filter paper, and they were then allowed to air dry. Micrographs of rE2/E3BP and tE2/E3BP were recorded at sample concentrations of 19.7 and 32 nM, either at high magnifications (90,000× to 120,000×) on a Gatan-Erlangshen-lens-coupled CCD camera or under low-electron-dose conditions at a magnification of 30,000× on Kodak S0163 film. Micrographs recorded on film were subsequently digitised at 5000 dots/in. using a Nikon Coolscan 4000 transparency scanner.

### Cryo-EM and image reconstruction

E3 used for cryo-EM experiments was expressed and purified as described elsewhere.^69^ rE2/E3BP and E3 were mixed at a ratio of 2:1 (rE2/E3BP:E3). It is to be noted that the ratio here refers only to the ratio of monomeric E3BP to dimeric E3 (E3BP:E3); therefore, 2:1 represents 12E3BP:6E3 (12E3BP of the rE2/E3BP core bound to 6E3). Images of rE2/E3BP and rE2/E3BP:E3 complexes in vitreous ice were obtained using an FEI F30 cryo-electron microscope operating at a magnification of 38,000× and at 300 kV. The negatives were captured on Kodak SO-163 film and scanned on a Zeiss SCAI scanner with a raster size of 7 μm, giving a pixel size of 1.8 Å at the specimen. The images were CTF corrected on a per-micrograph basis using the EMAN suite program Ctfit, and subsequently normalised and binned to a pixel size of 3.6 Å using the EMAN program Proc2d.^70^ Nine hundred eighty-two images from 16 micrographs were used for the rE2/E3BP reconstruction, and 172 images from 9 micrographs were used for the rE2/E3BP:E3 reconstruction; all particles were picked interactively using Boxer from the EMAN suite.^70^ Reconstructions were initially determined using the program IMAGIC,^71^ assuming dodecahedral symmetry, and then refined in SPIDER,^72^ making the same assumption. The reconstructions reported here have resolutions of 18.3 Å for the rE2/E3BP map and 33.0 Å for the rE2/E3BP:E3 map. The Fourier shell correlation criterion for resolution (Fourier shell correlation = 0.5) was used. Maps were filtered, and the SAS model was prepared for comparison with them using the software WellMAP (J. F. F. Flanagan, IV and R.J.C.G., unpublished); this included inverting the hand of the scattering model since this produced a much better alignment of the regions of the structures extending from the PDC core surface. The maps have been deposited in the PDB Europe structure database for EM structures^b^ with accession codes EMD-1658 and EMD-1659, and will be released for download on publication.

### Homology modelling and superimposition of cores

A homology model of the recombinant human truncated E2 (tE2) was obtained from the SWISS-MODEL server^c^.^68–76^ The crystal structure of *B*. *stearothermophilus* E2 (PDB ID 1B5S)^28^ was used as template, and the model was submitted via project mode in the program Swiss-PdbViewer^d^.^74^ The human tE2 model obtained from SWISS-MODEL was a pentamer, and the complete oligomeric 60-mer was built using crystallographic symmetry with the program PyMOL (Delano Scientific, USA). All structures were visualised using the programs VMD^e^,^77^ Swiss-PdbViewer^74^ and PyMOL (Delano Scientific). Superimposition of high-resolution atomic solution structures and low-resolution solution structures was performed using the program SUPCOMB,^55^ employing default parameters.

### Hydrodynamic modelling

The programs HYDRO^++^^53,54^ and SUPCW^78^ were used to calculate the hydrodynamic properties of atomic resolution structures. HYDRO^++^ is an improved version of an earlier program HYDRO,^54^ with enhanced calculations for rotational properties and intrinsic viscosities. Hydrodynamic parameters, including the anhydrous sedimentation coefficient *s*_0_ and the translational diffusion coefficient *D*_t_, were obtained by providing an HBM generated from atomic coordinates as an input to HYDRO^++^ and SUPCW. The GASBOR-generated ab initio models of rE2/E3BP and tE2/E3BP were converted into HBMs employing a 15-Å and 5.7-Å cubic grid using the AtoB algorithm^52^ implemented within SOMO,^79^ now part of the program ULTRASCAN^f^.^80^ The HBMs used for calculations with HYDRO^++^ were corrected to the exact volumes of rE2/E3BP and tE2/E3BP, obtained from calculations based on the molecular mass *M*, the partial specific volume *v̄* and Avogadro's number *N*_A_ (6.023 × 10^23^ mol^−^ ^1^). Alternatively, HBMs of rE2/E3BP and tE2/E3BP with no bead overlaps were generated using a 7-Å cubic grid with the program TRANS2VORONOI, an extended version of AtoB developed by M. Nöllmann (Centre de Biochimie Structurale Montpellier, France), and hydrodynamic computations were performed using the SUPCW subroutine of BEAMS.^78^

Only surface hydrodynamic hydration is “seen” by HBM programs such as HYDRO^++^; solvent entrapped in central cavities or in empty faces of icosahedra (e.g., in the case of PDC) does not contribute.^52^ As a consequence, the hydration factor applied to correct the computed anhydrous sedimentation coefficient is derived from a much lower level of hydration compared to the maximum possible value suggested by the frictional ratio.^81^ The level of hydration appropriate for correction of *s* for the architecturally comparable spherical hollow multi-protein apoferritin complex was found to be 0.2 g water/g protein.^52^ We could not justify choosing this exact value for correction of PDC sedimentation coefficients; therefore, a (similar) typical hydration of 0.4 g water/g protein was used.

Anhydrous sedimentation coefficients obtained from HYDRO^++^ and SUPCW were converted into their equivalent hydrated values by employing a conversion factor of 0.87 (derived from hydration of 0.4 g water/g protein using the relationship $F={\left(\overset{―}{v}/\left(\overset{―}{v}+\delta_{1}v_{1}^{0}\right)\right)}^{1/3}$, where δ_1_ is the hydration and *v*_1_^0^ is the specific volume of the solvent).^52^ All models obtained from AtoB and TRANS2VORONOI were visualised with one of several programs: PyMOL (Delano Scientific), RASMOL^g^^82^ or FreeWRL^h^. AtoB was used to construct HBMs from ab initio models obtained from SAS data and atomic resolution structures, while TRANS2VORONOI employed only the ab initio SAS models for HBM generation.

### CD spectroscopy

CD studies (far-UV and near-UV) were performed to assess the core stability of rE2/E3BP, rE2 and tE2/E3BP in PEBS100 buffer in the presence of denaturant (0–6 M GdmCl) at 25 °C. Experiments were conducted on a JASCO J-810 spectropolarimeter with a 1-nm bandwidth and with scan speeds and response times of 50 nm/min and 0.5 s for far-UV, and 10 nm/min and 2 s for near-UV. Quartz cells (path length, 0.5 and 0.02 cm) were used for near-UV and far-UV, covering the ranges 250–320 and 180–260 nm, respectively. Data were recorded at sample concentrations of 0.2 mg/ml (far-UV) and 0.9 mg/ml (near-UV) for rE2/E3BP, rE2 and tE2/E3BP in the presence of increasing amounts of GdmCl. GdmCl concentration was accurately measured via refractometry prior to each run. The extent of unfolding or dissociation in the presence of increasing denaturant was monitored at 222 nm (far-UV) and 285 nm (near-UV) by calculating the percent total change in ellipticity. Additionally, the Gibbs free energy at infinite dilution Δ*G*^0^ of the unfolding reactions under standard conditions of temperature and pressure was also computed. As data obtained from far-UV and near-UV CD showed similar results, only near-UV CD data are presented here, unless stated otherwise.

### Tryptophan fluorescence spectroscopy

Changes in tryptophan fluorescence were monitored for rE2, rE2/E3BP and tE2/E3BP samples in PEBS100 buffer during chemical denaturation in the presence of increasing concentrations of GdmCl (0–6 M). The cores (0.2 mg/ml) were excited at 295 nm, and fluorescence emissions were monitored over the spectral range 310–450 nm using a Perkin-Elmer L55OB spectrophotometer at 25 °C. Quartz cells (path length, 1 cm) were used, and data were analysed with software provided by the manufacturer.

## Figures and Tables

**Table 1:** Hydrodynamic parameters for recombinant cores derived from SV data

Core	*M* (Da)	*v̄* (ml/g)	*s*_20,w_^0^ (S)	*f*/*f*_0_	*R*_0_ (Å)	*R*_s_ (Å)	*D*_s_ (Å)	*D*_t_ (cm^2^/s)
rE2/E3BP	3,551,100	0.744	29.3	2.69	102	273	546	8.10 × 10^−^ ^8^
rE2	3,741,780	0.744	29.3	2.79	103	288	576	7.68 × 10^−^ ^8^
tE2/E3BP	1,671,348	0.746	27.5	1.73	791	137	274	1.569 × 10^−^ ^7^

*M* is the molecular mass based on amino acid composition; *v̄* is the calculated partial specific volume at 20 °C; *s*_20,w_^0^ is the standardised experimental sedimentation coefficient at infinite dilution; *R*_0_ is the radius of an anhydrous sphere of mass and specific volume equivalent to the core in question; *R*_s_ is the hydrodynamic or Stokes radius; *D*_s_ is the diameter of the particle obtained from the Stokes radius; and *D*_t_ is the translational diffusion coefficient.

## Footnotes

† http://expasy.org/tools/protparam.html

‡ http://www.jphilo.mailway.com/

§ http://www.rasmb.bbri.org/rasmb/windows/uconn_uaf/

‖ http://www.embl-hamburg.de/ExternalInfo/Reasearch/Sax/software.html

¶ http://www.ill.eu/instruments-support/instruments-groups/groups/lss/grasp/home/

^**a**^ http://www.scotgrid.ac.uk/

^**b**^ http://www.ebi.ac.uk/pdbe

^**c**^ http://swissmodel.expasy.org/

^**d**^ http://www.expasy.org/spdbv/

^**e**^ http://www.ks.uiuc.edu/Research/vmd/

^**f**^ http://www.ultrascan.uthscsa.edu/

^**g**^ http://openrasmol.org

^**h**^ http://freewrl.sourceforge.net
